# Long Non-Coding RNA Modulation of VEGF-A during Hypoxia

**DOI:** 10.3390/ncrna4040034

**Published:** 2018-11-20

**Authors:** Tiina Nieminen, Tristan A. Scott, Feng-Mao Lin, Zhen Chen, Seppo Yla-Herttuala, Kevin V. Morris

**Affiliations:** 1 The Center for Gene Therapy, Beckman Research Institute, City of Hope, Duarte, CA 91010, USA; timaniem@gmail.com (T.N.); trscott@coh.org (T.A.S.); 2 Department of Biotechnology and Molecular Medicine, A.I. Virtanen Institute for Molecular Sciences, University of Eastern Finland, P.O. Box 1627, FI-70211 Kuopio, Finland; seppo.ylaherttuala@uef.fi; 3 Department of Diabetes Complications and Metabolism, Beckman Research Institute, City of Hope, Duarte, CA 91010, USA; flin@coh.org (F.-M.L.); zhenchen@coh.org (Z.C.); 4 Heart Center and Gene Therapy Unit, Kuopio University Hospital, FI-70211 Kuopio, Finland

**Keywords:** vascular endothelial growth factor A (VEGF-A), long non-coding RNA (lncRNA), transcriptional activation, antisense RNA

## Abstract

The role and function of long non-coding RNAs (lncRNAs) in modulating gene expression is becoming apparent. Vascular endothelial growth factor A (VEGF-A) is a key regulator of blood vessel formation and maintenance making it a promising therapeutic target for activation in ischemic diseases. In this study, we uncover a functional role for two antisense VEGF-A lncRNAs, *RP1-261G23.7* and *EST AV731492*, in transcriptional regulation of *VEGF-A* during hypoxia. We find here that both lncRNAs are polyadenylated, concordantly upregulated with *VEGF-A*, localize to the *VEGF-A* promoter and upstream elements in a hypoxia dependent manner either as a single-stranded RNA or DNA bound RNA, and are associated with enhancer marks H3K27ac and H3K9ac. Collectively, these data suggest that VEGF-A antisense lncRNAs, *RP1-261G23.7* and *EST AV731492*, function as *VEGF-A* promoter enhancer-like elements, possibly by acting as a local scaffolding for proteins and also small RNAs to tether.

## 1. Introduction

Over the last decade, the role of non-coding transcripts, both long and short forms, in controlling transcriptional and epigenetic states has become realized (reviewed in [1,2]). Early observations suggested that promoters are susceptible to small RNA directed transcriptional gene silencing and gene activation [3,4]. Small RNA directed transcriptional activation has previously been observed with vascular endothelial growth factor A (*VEGF-A*) [4,5,6,7] and appears to be the result of the small RNA interacting with sense or antisense non-coding transcripts at the *VEGF-A* promoter [8]. Mechanistically, it appears that the small RNAs interact with transcripts at the targeted promoter to imbue the recruitment of various epigenetic remodeling complexes, resulting in transcriptional gene silencing or activation (reviewed in [9,10]).

Ischemic heart disease (IHD) consists of group of diseases where the supply of blood to the heart muscle is restricted causing chest pain or discomfort. The underlying mechanism behind IHD is the narrowing of the coronary arteries due to atherosclerotic plague buildup or acute occlusion. VEGF-A is one of the dominant inducers of blood vessel growth making it a promising therapeutic target for activating in treating IHD [11]. The activation of *VEGF-A* with small hairpin RNAs (shRNAs) has been found to ameliorate damage to the heart following IHD [6,12]. We set out here to further explore the network of non-coding RNAs involved in regulating the *VEGF-A* promoter. Specifically, two antisense long non-coding RNAs (lncRNAs) were interrogated, as antisense lncRNAs have been observed to epigenetically modulate genes such as *PTEN* [13,14], *P21* [15], *P15* [16] as well as several others (reviewed in [10,17]). We find here that *VEGF-A* and the *VEGF-A* associated antisense transcripts are concordantly linked and that the antisense transcripts function locally at the promoter as enhancer elements to modify *VEGF-A* expression. 

## 2. Results

### 2.1. Antisense Transcript Expression Is Concordantly Upregulated with Vascular Endothelial Growth Factor A in Hypoxia

**Figure 1:**
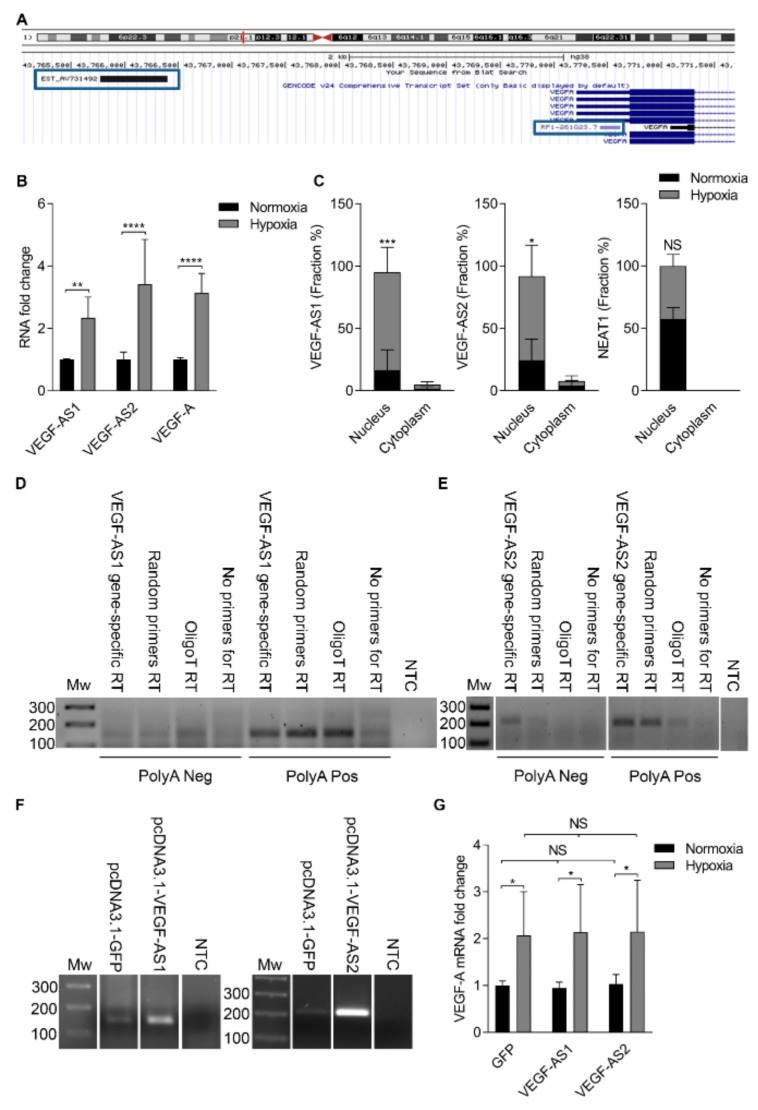
The expression of both vascular endothelial growth factor A (VEGF-A) promoter associated antisense long non-coding RNAs (lncRNAs) is upregulated in hypoxia. (**A**) A schematic is shown depicting the location of *RP1-261G23.7* (*VEGF-AS1*) and *EST AV731492* (*VEGF-AS2*) in the human genome relative to the *VEGF-A* gene; (**B**) fold change in *VEGF-AS1*, *VEGF-AS2* and spliced *VEGF-A* expression levels in EA.hy926 cells ± hypoxia as determined by quantitative reverse transcription -polymerase chain reaction (qRT-PCR) and standardized to β-2-microglobulin (*B2M*). The data are presented as mean ± standard deviation (SD) (*n* = 3 independent experiments). Significance was measured by two-way analysis of variance (ANOVA). ** *p* < 0.01, **** *p* < 0.0001; (**C**) qRT-PCR analysis of *VEGF-AS1*, *VEGF-AS2* and nuclear paraspeckle assembly transcript 1 (*NEAT1*) expression in subcellular fractions from EA.hy926 cells ± hypoxia, plotted as percentages in association with nucleus and cytoplasm. The data are presented as mean ± SD (*n* = 3 independent experiments). Significance was measured by two-way ANOVA. * *p* < 0.5, *** *p* < 0.001; (**D**) RT-PCR analysis of *VEGF-AS1* expression in polyA depleted and polyA positive fractions in EA.hy926 cells. The data are representative of two independent experiments; (**E**) RT-PCR analysis of *VEGF-AS2* expression in polyA depleted and polyA positive fractions in EA.hy926 cells. The data are representative of two independent experiments; (**F**) Over-expression of *VEGF-AS1* and *VEGF-AS2* in EA.hy926 cells 72 h after transfection relative to the pcDNA3.1-GFP control; (**G**) Fold change in *VEGF-A* expression in normoxic and hypoxic EA.hy926 cells 72 h after *VEGF-AS1* and *VEGF-AS2* transfections relative to the control pcDNA3.1-GFP as determined by qRT-PCR and standardized to *B2M*. The data are presented as mean ± SD (*n* = 3 independent experiments). Significance was measured by two-way ANOVA. * *p* < 0.05. NS, non-significant; RT, reverse transcription; NTC, no template control; Mw, molecular weight; GFP, green fluorescent protein.

Assessment of the University of California Santa Cruz (UCSC) genome browser indicated that two lncRNAs, *RP1-261G23.7* (*VEGF-AS1*) and *EST AV731492* (*VEGF-AS2*), appear antisense to the *VEGF-A* promoter and may therefore function in the transcriptional and epigenetic regulation of *VEGF-A* (Figure 1A). *RP1-261G23.7* is 188 nucleotides long and overlaps the 5′ untranslated region (UTR) of *VEGF-A*. *EST AV731492* is putatively 680 nucleotides long and is located more than 4 kb upstream of *VEGF-A* transcription start site (TSS). Vascular endothelial growth factor A is known to be activated following hypoxia. To determine to what extent these lncRNAs are also affected by hypoxia, we induced hypoxia in human EA.hy926 cells. The expression of both transcripts was found to be activated along with *VEGF-A* following hypoxia in EA.hy926 cells as determined by quantitative reverse transcription-polymerase chain reaction (qRT-PCR) (Figure 1B). To characterize the transcripts further, a subcellular fractionation was performed on EA.hy926 cells. These data demonstrated that both *VEGF-AS1* and *VEGF-AS2* transcripts were predominantly expressed in the nucleus in a hypoxia-dependent manner (Figure 1C). A nuclear transcript nuclear paraspeckle assembly transcript 1 (*NEAT1*) was used as a positive control [18], which was found to be equally expressed in both normoxia and hypoxia treated samples (Figure 1C). To determine if the *VEGF-A* associated antisense lncRNAs were poly-adenylated, EA.hy926 cells were split into a poly-A positive and negative fractions. Both *VEGF-AS1* and *VEGF-AS2* appeared to be poly-adenylated (Figure 1D,E). Collectively, these data suggest, similar to previous observations [8], that both lncRNAs are concordantly activated in the nucleus with *VEGF-A* during hypoxia.

### 2.2. Antisense Transcripts Modify Vascular Endothelial Growth Factor A Expression

**Figure 2:**
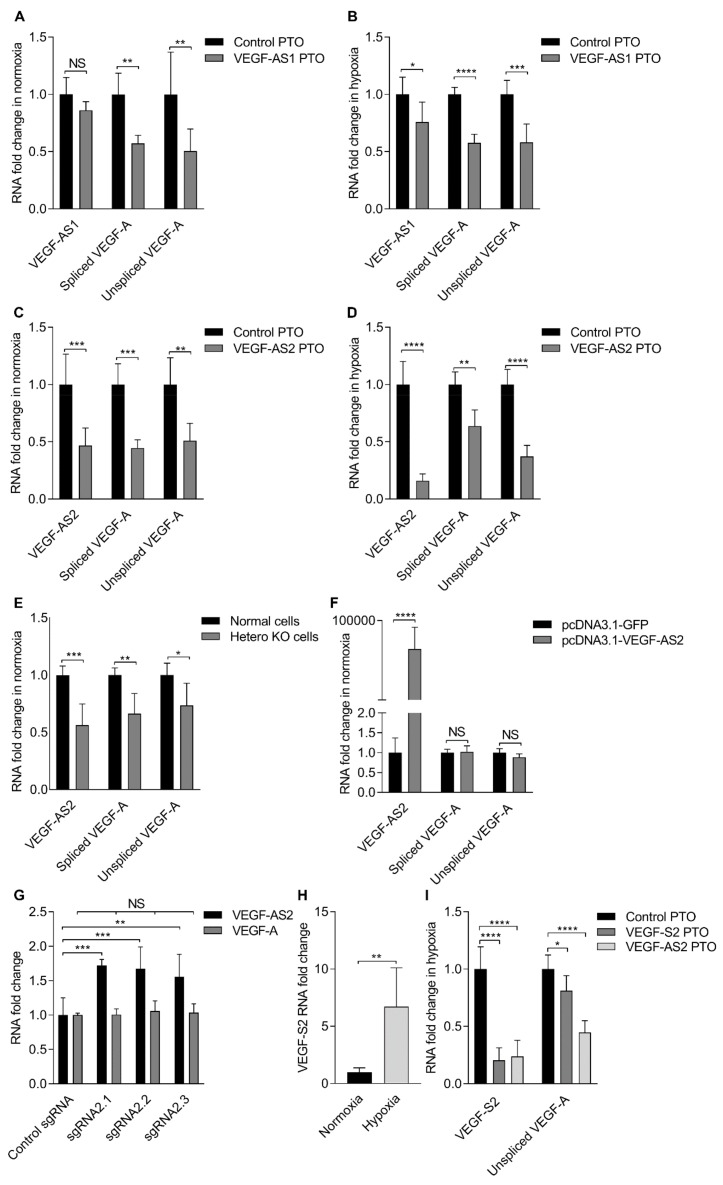
Repression of *VEGF-A* promoter associated antisense lncRNAs results in the downregulation of *VEGF-A* expression. (**A**) Fold change in *VEGF-AS1* and spliced or unspliced *VEGF-A* expression levels in normoxic EA.hy926 cells 48 h after antisense phosphorothioate oligonucleotides (PTO) transfections as determined by qRT-PCR and standardized to *B2M*. The data are presented as mean ± SD (*n* = 3 independent experiments). Significance was measured by two-way ANOVA. ** *p* < 0.01; (**B**) fold change in *VEGF-AS1* and spliced or unspliced *VEGF-A* expression levels in hypoxic EA.hy926 cells 48 h after antisense PTO transfections as determined by qRT-PCR and standardized to *B2M*. The data are presented as mean ± SD (*n* = 3 independent experiments). Significance was measured by two-way ANOVA. * *p* < 0.05, *** *p* < 0.001, **** *p* < 0.0001; (**C**) Fold change in *VEGF-AS2* and spliced or unspliced *VEGF-A* expression levels in normoxic EA.hy926 cells 48 h after antisense PTO transfections as determined by qRT-PCR and standardized to *B2M*. The data are presented as mean ± SD (*n* = 3 independent experiments). Significance was measured by two-way ANOVA. ** *p* < 0.01, *** *p* < 0.001; (**D**) Fold change in *VEGF-AS2* and spliced or unspliced *VEGF-A* expression levels in hypoxic EA.hy926 cells 48h after antisense PTO transfections as determined by qRT-PCR and standardized to *B2M*. The data are presented as mean ± SD (*n* = 3 independent experiments). Significance was measured by two-way ANOVA. ** *p* < 0.01, **** *p* < 0.0001; (**E**) fold change in *VEGF-AS2* and spliced or unspliced *VEGF-A* expression levels in normal and knockout (KO) EA.hy926 cells as determined by qRT-PCR and standardized to *B2M*. The data are presented as mean ± SD (*n* = 2 independent experiments). Significance was measured by two-way ANOVA. * *p* < 0.05, ** *p* < 0.01, *** *p* < 0.001; (**F**) Fold change in *VEGF-AS2* and spliced or unspliced *VEGF-A* expression levels in KO EA.hy926 cells 72 h after *VEGF-AS2* transfections relative to the control pcDNA3.1-GFP as determined by qRT-PCR and standardized to *B2M*. The data are presented as mean ± SD (*n* = 2 independent experiments). Significance was measured by two-way ANOVA. **** *p* < 0.0001; (**G**) fold change in *VEGF-AS2* and spliced *VEGF-A* expression levels in EA.hy926 cells 48 h after transfections as determined by qRT-PCR and standardized to *B2M*. The data are presented as mean ± SD (*n* = 2 independent experiments). Significance was measured by two-way ANOVA. ** *p* < 0.01, *** *p* < 0.001; (**H**) Fold change in *VEGF-S2* expression levels in normoxic and hypoxic EA.hy926 cells as determined by qRT-PCR and standardized to *B2M*. The data are presented as mean ± SD (*n* = 3 independent experiments). Significance was measured by two-tailed unpaired t test. ** *p* < 0.01; (**I**) Fold change in *VEGF-S2* and unspliced *VEGF-A* expression levels in hypoxic EA.hy926 cells 48 h after antisense PTO transfections as determined by qRT-PCR and standardized to *B2M*. The data are presented as mean ± SD (*n* = 3 independent experiments). Significance was measured by two-way ANOVA. * *p* < 0.05, **** *p* < 0.0001.

The effects of over-expressing and knocking-down both *VEGF-A* associated lncRNAs were assessed in EA.hy926 cells. The over-expression of *VEGF-AS1* and *VEGF-AS2* in EA.hy926 cells (Figure 1F) did not have any notable effect on *VEGF-A* expression (Figure 1G) neither in normoxia or hypoxia. In hypoxia, *VEGF-A* expression was upregulated, but there were no additional changes after *VEGF-AS1* or *VEGF-AS2* transfections (Figure 1G). However, the repression of the *VEGF-A* associated lncRNAs resulted in a more profound effect on *VEGF-A* expression (Figure 2A–D). Antisense phosphorothioate oligonucleotides (PTOs) were used to knock-down lncRNA expression and the effects on both spliced and unspliced *VEGF-A* RNA expression were assessed in both normoxic and hypoxic cells. In both PTO treated normoxic (Figure 2A,C) and hypoxic cells (Figure 2B,D), the repression of both lncRNAs appeared to result in a down-modulation of *VEGF-A*, with *VEGF-AS2* showing a particularly robust effect on *VEGF-A* expression (Figure 2C,D). The antisense transcript 1 knock-down and its effects were also analyzed in PC-3 cells (Supplementary Materials Figure S2). To verify the effect of *VEGF-AS2* on *VEGF-A*, clustered regularly interspaced short palindromic repeats (CRISPR/Cas9) technology was used to knockout (KO) one copy of the *VEGF-AS2* in EA.hy926 cells (Figure 2E,F). Both the spliced and unspliced *VEGF-A* RNA levels were shown to be downregulated in these heterozygous KO cells (Figure 2E). Similar to observations in normal EA.hy926 cells, *VEGF-AS2* over-expression did not have any effect on *VEGF-A* expression in KO cells (Figure 2F). These data suggest that both *VEGF-AS1* and *VEGF-AS2* transcripts regulate *VEGF-A* expression in cis and notable not observable effects are seen when introducing these transcripts in trans. Considering these observations, we decided to explore endogenous *VEGF-AS2* upregulation approach, since over-expression from a plasmid did not have any observable effects on *VEGF-A* neither in normal or KO cells. We designed single guide RNA (sgRNAs) targeting the putative *VEGF-AS2* promoter and after co-transfection with an activating dCas9-VPR [19], *VEGF-AS2* levels were shown to increase 1.7-fold and did not have any notable effect on *VEGF-A* expression (Figure 2G). 

Previous studies, with small RNAs have observed that a promoter-associated sense transcript is required for RNA directed transcriptional gene silencing [20]. We also identified a sense transcript at the *VEGF-AS2* locus which, similar to the antisense transcript, exhibited increased expression during hypoxia (Figure 2H). The repression of the sense transcript with a PTO seemed to lead to a more modest *VEGF-A* downregulation than after *VEGF-AS2* knockdown (Figure 2I) suggesting that *VEGF-AS2* is the main modulator of *VEGF-A* expression, though other uncharacterized transcripts may also contribute to this phenomenon. Interestingly, the antisense transcript knockdown also decreased the sense transcript expression (Figure 2I) indicating that the sense transcript expression is dependent on *VEGF-AS2*, and suggesting a possible role for sense transcription at the *VEGF-AS2* locus.

### 2.3. Antisense Transcrpits Localize at the Vascular Endothelial Growth Factor A Promoter as a Single-Stranded RNA or Bound to the DNA

**Figure 3:**
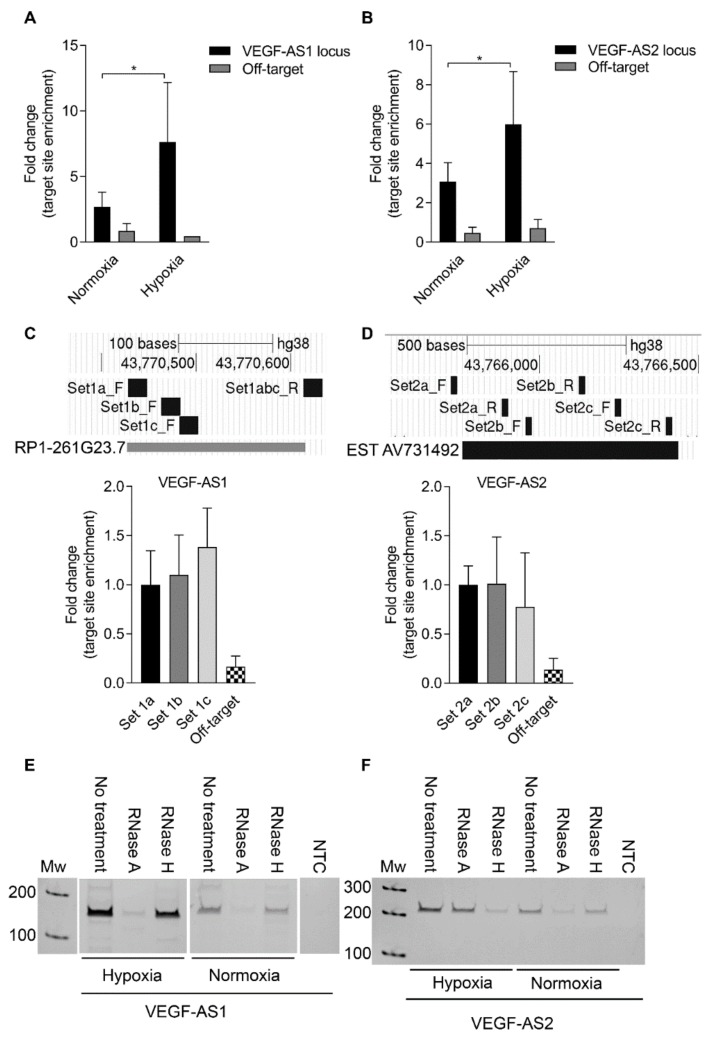
Both *VEGF-AS1* and *VEGF-AS2* localize to the *VEGF-A* promoter. (**A**) Fold change in *VEGF-AS1* target locus enrichment at the *VEGF-A* promoter in normoxic and hypoxic EA.hy926 cells as determined by quantitative polymerase chain reaction (qPCR) after pulldown with antisense oligonucleotides with 3′-Biotin modifications. Beads only control is set to be 1. The data are presented as mean ± SD and standardized to inputs (*n* = 3 independent experiments). Significance was measured by two-way ANOVA. * *p* < 0.05; (**B**) fold change in *VEGF-AS2* target locus enrichment at the *VEGF-A* promoter in normoxic and hypoxic EA.hy926 cells as determined by qPCR after pulldown with antisense oligonucleotides with 3′-Biotin modifications. Beads only control is set to be 1. The data are presented as mean ± SD and standardized to inputs (*n* = 3 independent experiments). Significance was measured by two-way ANOVA. * *p* < 0.05; (**C**) primer walking at the *VEGF-A* promoter. A qPCR analysis of *VEGF-AS1* localization at the *VEGF-A* promoter in EA.hy926 cells after pulldown with antisense oligonucleotides with 3′-Biotin modifications. The data are presented as mean ± SD and standardized to inputs (*n* = 2 independent experiments); (**D**) primer walking at the *VEGF-A* promoter. A qPCR analysis of *VEGF-AS2* localization at the *VEGF-A* promoter in EA.hy926 cells after pulldown with antisense oligonucleotides with 3′-Biotin modifications. The data are presented as mean ± SD and standardized to inputs (*n* = 2 independent experiments); (**E**) RT-PCR analysis of *VEGF-AS1* expression in hypoxic and normoxic EA.hy926 cells after pulldown with antisense oligonucleotides with 3′-Biotin modifications and treatments with RNase A and RNase H. The data are representative of three independent experiments; (**F**) RT-PCR analysis of *VEGF-AS2* expression in hypoxic and normoxic EA.hy926 cells after pulldown with antisense oligonucleotides with 3′-Biotin modifications and treatments with RNase A and RNase H. The data are representative of three independent experiments.

To determine to what extent the *VEGF-A* associated lncRNAs are actively interacting with the local *VEGF-A* promoter and associated chromatin, a chromatin isolation by RNA purification (ChIRP) assay was performed from EA.hy926 cells. Antisense oligonucleotides with 3′-biotin modifications targeting *VEGF-AS1* or *VEGF-AS2* were used to pulldown the RNA–DNA-protein complexes. Both *VEGF-AS1* and *VEGF-AS2* transcripts were shown to localize specifically to their corresponding homologous target sites at the *VEGF-A* promoter and upstream elements (Figure 3A,B and Supplementary Materials Figure S1). Fascinatingly, these interactions were increased during hypoxia. *VEGF-A* promoter tiling with multiple primer pairs, to better resolve those loci bound by the respective lncRNAs, verified the localization of *VEGF-AS1* and *VEGF-AS2* to the homology containing loci at the *VEGF-A* promoter and upstream elements (Figure 3C,D). Chromatin isolation by RNA purification complexes were also subjected to RNase A and RNase H treatments (Figure 3E,F). *VEGF-AS1* was shown to stay as a single-stranded RNA both in hypoxia and normoxia (Figure 3E), but interestingly, *VEGF-AS2* was found to be bound to the DNA in hypoxia (Figure 3F). These data imply that during hypoxia, more *VEGF-AS1* and *VEGF-AS2* transcripts are expressed resulting in the increased localization specifically at their sites of transcription, e.g., cis-functional. *VEGF-AS1* remains as a single-stranded RNA molecule in hypoxia but *VEGF-AS2* appears to form DNA–RNA hybrids at the upstream *VEGF-AS2* locus.

### 2.4. Enhancer Marks Are Associated with Antisense Transcripts

**Figure 4:**
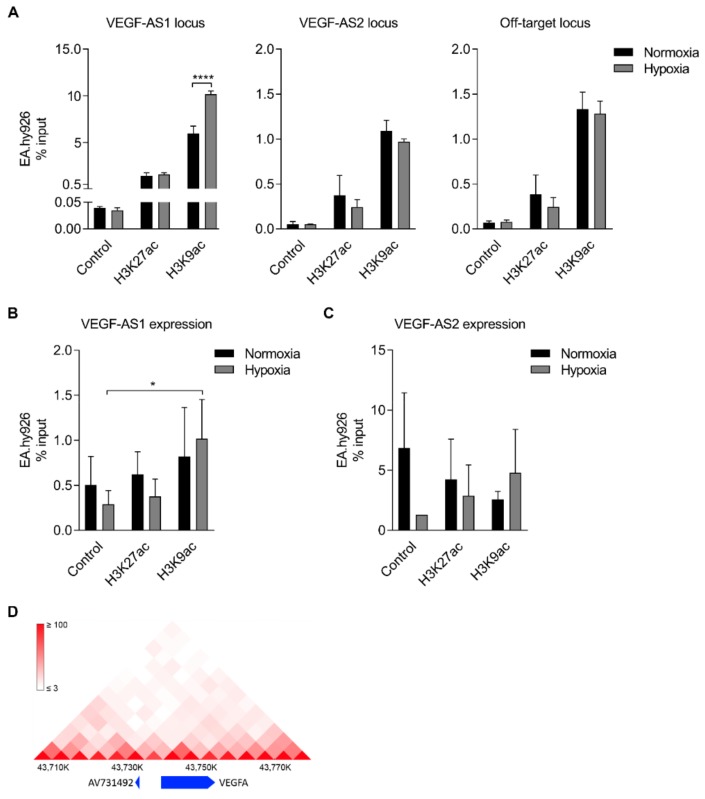
Strong enhancer marks are associated with *VEGF-AS1* and *VEGF-AS2*; (**A**) A qPCR analysis of H3K27ac and H3K9ac enrichment at the *VEGF-AS1*, *VEGF-AS2* and off-target loci in normoxic and hypoxic EA.hy926 cells. IgG is used as a control. The data are presented as mean ± SD and standardized to inputs (*n* = 3 independent experiments). Significance was measured by two-way ANOVA. **** *p* < 0.0001; (**B**) A qRT-PCR analysis of H3K27ac and H3K9ac association with *VEGF-AS1* in normoxic and hypoxic EA.hy926 cells. IgG is used as a control. The data are presented as mean ± SD and standardized to inputs (*n* = 3 independent experiments). Significance was measured by two-way ANOVA. * *p* < 0.05; (**C**) A qRT-PCR analysis of H3K27ac and H3K9ac association with *VEGF-AS2* in normoxic and hypoxic EA.hy926 cells. IgG is used as a control. The data are presented as mean ± SD and standardized to inputs (*n* = 2–3 independent experiments); (**D**) the prediction of physical contacts between the *VEGF-AS2* locus and the VEGF-A promoter in human umbilical vein endothelial cells (HUVECs). The figure was generated by using three-dimensional (3D) Genome Browser (http://biorxiv.org/content/early/2017/02/27/112268). The track around the *VEGF-A* gene loci was selected in 5 kilobase resolution in HUVECs with genome version HG19.

Acetylations at lysine residues of histone H3 have shown to be associated with enhancers and promoters of active genes [21]. In order to study the enhancer mark (H3K27ac and H3K9ac) enrichment at the *VEGF-AS1* and *VEGF-AS2* loci chromatin immunoprecipitation (ChIP) and RNA immunoprecipitation (RIP) assays were carried out from EA.hy926 cells. At the *VEGF-AS1* locus, both enhancer marks were shown to be enriched and H3K9ac enrichment was higher in hypoxic cells (Figure 4A). Some enhancer mark enrichment, although to a lower level, was also detected at the *VEGF-AS2* locus (Figure 4A). However, comparable amount of enrichment was found in the off-target locus, situated 9 kb upstream of *VEGF-A* (Figure 4A). H3K27ac and H3K9ac enhancer marks were also shown to be associated with the lncRNAs themselves (Figure 4B,C). Notably, during hypoxia, the association between H3K9ac histone mark and *VEGF-AS1* was evident and there was also a trend in the association between H3K9ac histone mark and *VEGF-AS2* in hypoxia. This trend was clearly seen in each independent experiment in hypoxic conditions, although the values somewhat differ. (Figure 4B,C). These results imply that both *VEGF-AS1* and *VEGF-AS2* function as hypoxia induced enhancer-associated factors, and that the *VEGF-AS1* locus per se seems to be a more potent enhancer. To further assess this notion, a chromatin interaction analysis in human umbilical vein endothelial cells (HUVECs) (Figure 4D) was carried out and suggested that *VEGF-AS2* locus might be interacting with another genomic locus at the *VEGF-A* promoter. However, since the distance is quite short, it is nearly impossible to distinguish between random events and functional direct contacts.

## 3. Discussion

Vascular endothelial growth factor A (VEGF-A) is a key player in blood vessel development and maintenance as well as in several pathological conditions such as cancer and cardiovascular diseases. However, the lack of understanding its transcriptional regulation is a major hurdle when it comes to developing new therapies for either decreasing or increasing blood vessel growth. Long non-coding RNAs (lncRNAs) have been shown to be potential candidates for targeted therapies due to their tissue and disease specificities, and some therapeutic lncRNA targets for angiogenesis have already been characterized (reviewed in [22]). We focused here on interrogating the function of two antisense lncRNAs that are associated with the *VEGF-A* promoter and upstream elements.

Both *VEGF-AS1* and *VEGF-AS2* transcripts were shown to be concordantly upregulated among *VEGF-A* during hypoxia. This has also been observed previously with other non-coding transcripts at the *VEGF-A* promoter in different cell types [8] suggesting that several *VEGF-A* promoter-associated non-coding transcripts respond to hypoxia similarly to *VEGF-A*, and might, therefore, be involved in *VEGF-A* regulation. Also, in this study, we identified a sense transcript at the *VEGF-AS2* locus that is responsive to hypoxia. To further characterize the antisense transcripts, we carried out subcellular fractionation, since the cellular localization of the lncRNA can provide clues to its function. Both *VEGF-AS1* and *VEGF-AS2* were shown to be highly nuclear-specific and the nuclear localization was increased in hypoxia supporting our hypothesis that these transcripts may be involved in transcriptional gene regulation. Previous observations with *trans* functional lncRNAs have shown direct localization of these transcripts to homology containing loci [13,23]. We also verified the localization of *VEGF-AS1* and *VEGF-AS2* to their site of transcription further strengthening their role in *VEGF-A* regulation. Our data also showed that both of the antisense transcripts appear to be poly-adenylated. The lncRNA for neuronal MYC (*MYCN*), *MYCNOS*, an antisense lncRNA that binds local chromatin sites in *cis* to regulate the expression of *MYCN*, is also poly-adenylated [24] and has an open reading frame coding for a bona-fide protein [25]. However, the lack of long open reading frames in *VEGF-AS1* and *VEGF-AS2* as well as using the PhyloCSF [26] algorithm suggest that these transcripts do not have protein-coding potential. 

Functional studies with transcript over-expression and knockdown revealed a cis regulatory mechanism for *VEGF-AS1* and *VEGF-AS2*. Over-expression of the antisense transcripts did not have any effects on *VEGF-A* levels neither in normal or heterozygous KO cells, but transcript knockdown also reduced *VEGF-A* expression suggesting that *VEGF-AS1* and *VEGF-AS2* function locally, in *cis*, at their site of transcription in order to control concordantly the expression of their neighboring gene, *VEGF-A*. The modest increase in endogenous *VEGF-AS2* levels after CRISPR/dCas9 activation was not enough to increase *VEGF-A* levels indicating that more *VEGF-AS2* is needed before any effects on *VEGF-A* can be detected. *VEGF-AS1* knockdown with PTOs was not as successful as *VEGF-AS2* knockdown, however, even the modest knockdown was shown to reduce *VEGF-A* levels. It is possible that *VEGF-AS1* was not degraded efficiently enough, but, nevertheless, its function was disturbed. Bidirectional transcription has been shown to take place in human cells and examples in the literature show natural antisense transcripts regulating the protein-coding genes in *cis* either discordantly or concordantly by recruiting chromatin modifying complexes and transcription factors at the promoters of their corresponding sense mRNA (reviewed in [17,27]). *VEGF-AS1* seems to fit well into this picture. During hypoxia, *VEGF-AS1* transcription and enhancer mark association increase, both at the *VEGF-AS1* locus overlapping the 5′ UTR of *VEGF-A* and with the *VEGF-AS1* itself, indicating that this single-stranded RNA functions as a *cis*-acting enhancer-like RNA, possibly by recruiting epigenetic remodeling proteins and guide them to the *VEGF-A* promoter to increase transcription. 

Interestingly, *VEGF-AS2* seems to regulate *VEGF-A* in a different way. During hypoxia, *VEGF-AS2* was shown to form an RNA–DNA hybrid, i.e., an R-loop. In previous studies, R-loops have been shown to form between lncRNAs and DNA, and the involvement of R-loops in transcriptional regulation by lncRNAs has been shown as well [28,29] and reviewed in [30]. This implies that the R-loop structure could maintain chromatin in an open state at the *VEGF-AS2* locus promoting regulatory protein binding. *VEGF-AS2* is a mono-exonic intronless transcript and interestingly, it was recently shown that intronless transcripts are more prone to accumulate R-loop structure than their intron-containing counterparts [31]. However, since *VEGF-AS2* transcript is localized approximately 4 kb upstream of the *VEGF-A* transcriptional start site, DNA looping might occur ultimately bringing the *VEGF-AS2* locus closer to the *VEGF-A* promoter. Previous published observations of lncRNAs [32,33,34] also support a chromatin looping model. The more modest enhancer mark enrichment at the *VEGF-AS2* locus compared to the *VEGF-AS1* locus indicates that *VEGF-AS2* locus per se is not a strong enhancer. In addition, our off-target locus showed similar amounts of enrichment implying that this level of enrichment might not be specific for an enhancer. However, the possible interaction of *VEGF-AS2* with another genomic locus at the active promoter area may explain its enhancer-like functions, as enhancer marks were nevertheless shown to be associated with *VEGF-AS2* in hypoxic cells. Thus, during hypoxia, *VEGF-AS2* transcription increases and the transcript binds to DNA. This may lead to an opening up of the chromatin making regulatory factors, including chromatin modifying proteins, more accessible to bind. *VEGF-AS2* may enhance chromatin looping bringing the possibly bound proteins and other factors, including the sense non-coding transcript we identified, into physical proximity with the *VEGF-A* promoter to drive histone H3 lysine acetylations and gene transcription. The concordant regulation of protein-coding genes by lncRNAs is a less well-known phenomena than discordant and it is not known what dictates between these two [27]. It is therefore highly interesting that *VEFG-A* seems to be under concordant regulation by both *VEGF-AS1* and *VEGF-AS2* and that this mechanism of action appears to be the result of the lncRNA acting in enhancer like functions.

Previous observations by Li et al. demonstrated that *VEGF-A* transcriptional activation can be induced by small double-stranded RNAs (dsRNAs) [4]. Later studies with different small activating RNAs have also shown *VEGF-A* activation [5,6,7,8] and one suggested mechanism of action has been that *VEGF-A* promoter-directed ncRNAs interact with promoter-associated sense or antisense transcripts, and these small activating RNAs have also been shown to recruit histone modifications at their target chromosomal loci. Indeed, binding of small RNAs to promoter-associated antisense transcripts has been observed previously with other genes [35,36,37,38]. Based on our current study, it is likely that *VEGF-AS1* and *VEGF-AS2* could form scaffolds for promoter-targeted ncRNAs, though future studies are required to verify this notion. Since short and long non-coding transcripts are poorly conserved between species and often times show tissue and cell-specificity, it is highly important to study the interactions in the context of same species and cell line and difficult to draw conclusions between various species, e.g., mouse and human. 

In conclusion, we have for the first time studied two *VEGF-A* promoter-associated antisense lncRNA transcripts and determined their functional role in *VEGF-A* transcriptional regulation. We show here that (1) *VEGF-AS1* and *VEGF-AS2* are concordantly upregulated with *VEGF-A* in hypoxia and localize to the *VEGF-A* promoter and upstream elements in a hypoxia-dependent manner, (2) *VEGF-AS1* and *VEGF-AS2* regulate *VEGF-A* expression and the transcripts act in *cis* on their site of transcription, (3) *VEGF-AS2* forms an R-loop in hypoxia and might also loop the chromatin closer to the *VEGF-A* promoter, and (4) *VEGF-AS1* and *VEGF-AS2* are associated with enhancer marks in hypoxia and they may act as a local scaffolding for proteins and small RNAs to tether. Targeting these transcripts or those proteins or ncRNAs which interact with them, may prove useful in strategies to enhance *VEGF-A* expression, in diseases resulting from oxygen deficiency. In addition, knocking down the lncRNAs could be utilized as a treatment strategy for diseases suffering from excess vascularization.

## 4. Materials and Methods 

### 4.1. Expression Vectors and Antisense Oligonucleotides

The sequences of antisense lncRNAs (*RP1-261G23.7*, *VEGF-AS1* and *EST AV731492*, *VEGF-AS2*, Supplementary Materials Table S1) were ordered as gBlocks from Integrated DNA Technologies (IDT, Coralville, IA, USA) and subcloned into pcDNA3.1 (+) (Thermo Fisher Scientific, Waltham, MA, USA) using EcoRV (NEB) and XbaI (NEB) restriction sites. pcDNA3.1-GFP was used as a control. SgRNAs targeting *VEGF-AS2* putative promoter were designed (Supplementary Materials Table S2), ordered from IDT and subcloned into pcDNA3.1-H1sgRNA described in [39]. SgRNA targeting alpha-1 antitrypsin (target site does not have a PAM sequence) was used as a control (Supplementary Materials Table S2). SP-dCas9-VPR was obtained from Addgene (Addgene plasmid #63798) [19]. Antisense PTOs and antisense oligonucleotides with 3′-Biotin modification targeting *VEGF-AS1* and *VEGF-AS2* were designed with Sfold software or Biosearch Technologies’ Stellaris FISH Probe Designer, respectively, and ordered from IDT (Supplementary Materials Table S2). In PTO experiments, miRN367 targeted [40,41] antisense PTO was used as a control (Supplementary Materials Table S2).

### 4.2. Cell Culture and Transfections

EA.hy926 cell line was cultured in Dulbecco’s modified eagle’s medium (DMEM, Sigma-Aldrich, St Louis, MO, USA) and PC-3 cell line in Roswell park memorial institute medium (RPMI) (Sigma-Aldrich) supplemented with 10% fetal bovine serum, 100 units/mL penicillin and 100 μg/mL streptomycin. For transfections, cells were plated in 12-well plates (1–2.5 × 105 cells/well). Twenty-four hours later, cells were transfected with Lipofectamine 3000 (Thermo Fisher Scientific) according to the manufacturer’s instructions, with 500 ng of DNA or 50 nM PTO. GasPak™ 100 System (BD) was used for hypoxia experiments. For the transfections, pcDNA3.1-VEGF-AS1 and pcDNA3.1-VEGF-AS2 were used to study the effects of over-expression of the lncRNAs, and antisense PTOs were used to knockdown endogenous lncRNA expression. For endogenous *VEGF-AS2* upregulation, cells were co-transfected with pcDNA3.1-H1sgRNAs and pcDNA3.1-dCas9-VPR. 48–72 h after transfections, cells were washed with phosphate buffered saline (PBS) and harvested into homogenization solution (Maxwell^®^ RSC simplyRNA Cells Kit, Promega, Madison, WI, USA). Alternatively, cells were subjected to 8 h hypoxia prior to harvesting.

### 4.3. Reverse Transcription and Quantitative Polymerase Chain Reaction

RNA was extracted with Maxwell^®^ RSC simplyRNA Cells Kit (Promega) and Maxwell^®^ RSC instrument (Promega) or with TRIzol Reagent (Thermo Fisher Scientific) according to manufacturer’s instructions. Briefly, after homogenizing the sample with TRIzol, chloroform was added and RNA was precipitated from the aqueous layer with isopropanol. The precipitated RNA was washed with ethanol to remove impurities. The amounts and purity of total RNAs were measured with NanoDrop ND-1000 Spectrophotometer (NanoDrop Technologies Inc., Wilmington, DE, USA). 0.1–1 µg of RNA was reverse transcribed into cDNA using either RT primer mix (QuantiTect Reverse Transcription Kit, Qiagen, Hilden, Germany) or gene-specific primers (IDT, Supplementary Materials Table S2) and Quantiscript Reverse Transcriptase (QuantiTect Reverse Transcription Kit, Qiagen). Quantitative measurements of RNA levels were done using KAPA SYBR^®^ FAST qPCR Master Mix (KAPA Biosystems, Wilmington, MA, USA) with the LightCycler^®^ 96 Instrument (Roche, Basel, Switzerland). Cycling parameters were 95 °C for 3 min, 40 cycles of 95 °C (3 s), and 60 °C (30 s) followed by a melting curve analysis. Various primer sets were used for quantitative polymerase chain reaction (qPCR) analysis (Supplementary Materials Table S2). Amplification of β-2-microglobulin (*B2M*) was used as an endogenous control to standardize the amount of RNA in each sample isolated from cell culture.

### 4.4. Subcellular Fractionation

EA.hy926 cells were separated into nuclear and cytoplasmic fractions using the protocol described by [42]. Briefly, cells were harvested and lysed with hypotonic lysis buffer (10 mM Tris (pH 7.5), 10 mM NaCl, 3 mM MgCl_2_, 0.3% NP-40, 10% glycerol) supplemented with with Halt™ Protease Inhibitor Cocktail (Thermo Fisher Scientific) and SUPERase In™ RNase Inhibitor (Thermo Fisher Scientific) to generate cytoplasmic extract and nuclei. From each fraction, protein or RNA can be isolated. For RNA isolation, nuclei were washed and directly lysed by incubation with TRIzol reagent (Thermo Fisher Scientifc). RNA in the cytoplasmic fraction was precipitated with 3 M sodium acetate (pH 5.5) and extracted with TRIzol. Reverse transcription was performed using either gene-specific primers or RT primer mix (QuantiTect Reverse Transcription Kit, Qiagen (Supplementary Materials Table S2). 

### 4.5. Polyadenylation (PolyA) Study

After total RNA extraction, the mRNA transcriptome was separated from the non-coding RNA population with Dynabeads™ mRNA Purification Kit (Thermo Fisher Scientific) according to manufacturer’s instructions. Same amount of polyA negative or polyA positive RNA fractions were subjected to reverse transcription with either gene-specific primers (Supplementary Materials Table S2), RT primer mix (QuantiTect Reverse Transcription Kit, Qiagen) or OligoT primers, followed by a PCR analysis with Q5^®^ Hot Start High-Fidelity Master Mix (NEB, Ipswich, MA, USA) and 0.5 µM of primers (Supplementary Materials Table S2).

### 4.6. Heterozygous VEGF-AS2 Knockout Cell Line

Clustered regularly interspaced short palindromic repeats (CRISPR/Cas9) technology was used to KO one copy of *VEGF-AS2* from EA.hy926 cells. Single guide RNA (sgRNAs) were designed and subcloned into pSpCas9(BB)-2A-GFP (PX458) (Addgene plasmid #48138) and pU6-(BbsI)_CBh-Cas9-T2A-mCherry (Addgene plasmid #64324), targeting 5′ and 3′ regions of *VEGF-AS2*, respectively. The sgRNAs were tested for the cutting efficiency using the Guide-it Mutation Detection Kit (Takara Bio USA, Mountain View, CA, USA). The best pair of sgRNAs, PX458-AV5C2 and pU6-(BbsI)_CBh-Cas9-T2A-mCherry-AV3C2 was selected for generating the KO cell line (for sgRNA sequences, see Supplementary Materials Table S2). According to the sgRNA target sequences, the donor template, pAV-DT-STD was constructed with 178 bp and 75 bp of 5′ and 3′ homology directed repair arms, respectively. The selection marker, PGK driven Puromycin resistance gene, and three SV40 polyadenylation signal repeats were also cloned in between the two arms. EA.hy926 cells were co-transfected with pX458-AV5C2, pU6-(BbsI)_CBh-Cas9-T2A-mCherry-AV3C2 and pAV-DT-STD using Lipofectamine 3000 (Thermo Fisher Scientific). The transfected cells were treated with 5 µg/mL of Puromycin after 48 h of transfection for two weeks to select the donor template inserted clones. The clone genotyping was confirmed by PCR. 

### 4.7. Chromatin Isolation by RNA Purification (ChIRP)

Fifteen million EA.hy926 cells were used per ChIRP sample. Cells were cross-linked with 1% formaldehyde at 4 °C for 10 min, followed by the addition of glycine to a final concentration of 0.125 M at 4 °C for 10 min. Cells were lysed in cell lysis buffer (5 mM PIPES, 85 mM KCl, 0.5% NP40) supplemented with Halt™ Protease Inhibitor Cocktail (Thermo Fisher Scientific) and SUPERase In™ RNase Inhibitor (Thermo Fisher Scientific) at 4 °C for 10 min, followed by lysis in nuclei lysis buffer (50 mMTris-HCl (pH 8), 10 mM ethylenediaminetetraacetic acid (EDTA), 1% sodium dodecyl sulfate (SDS)) supplemented with Halt™ Protease Inhibitor Cocktail and SUPERase In™ RNase Inhibitor at 4 °C for 20 min. Cell lysate was sonicated with a Bioruptor (Diagenode, Denville, NJ, USA) using pulse interval 30 s ON and 30 s OFF and a total of 30 cycles. Sonicated chromatin size was verified to be 200–500 bp before continuing. ChIRP assay was performed as described by [43,44]. Briefly, chromatin and antisense oligonucleotides with 3′-Biotin modifications were allowed to hybridize at 37 °C overnight. Dynabeads^®^ MyOne Streptavidin C1 magnetic beads (Thermo Fisher Scientific) were added to the mixture and after incubation at 37 °C for 2 h, beads were washed five times with wash buffer (2× SSC, 0.5% SDS supplemented with Halt™ Protease Inhibitor Cocktail) and elution was done with biotin competition (12.5 mM biotin, 7.5 mM HEPES (pH 7.5), 75 mM NaCl, 1.5 mM EDTA, 0.15% SDS, 0.075% sarkosyl, 0.02% sodium deoxycholate) at room temperature for 20 min and at 65 °C for 10 min. For DNA isolation, elute was treated with 0.5 M NaCl, 50 µg/mL RNase A (Thermo Fisher Scientific) and 100 µg/mL Proteinase K (NEB) at 65 °C overnight. Alternatively, DNA was eluted with RNase A (10 μg/mL) and RNase H (2 units) (Thermo Fisher Scientific) for 30 min at 37 °C and Proteinase K treatment was done at 50 °C for 45 min. DNA was purified with QIAquick PCR Purification Kit (Qiagen) and VEGF-AS1 and VEGF-AS2 localization was analyzed by qPCR (Supplementary Materials Table S2). For RNase A and RNase H treatments, streptavidin bead–RNA–DNA complexes were exposed to RNAse A (10 μg/mL) or RNase H (2 units) after the final wash step for 30 min at 37 °C. The samples were then heat inactivated (95 °C for 10 min for RNase A and 65 °C for 20 min for RNase H). All RNA samples were heated at 95 °C for 10 min and RNA was extracted with TRIzol (Thermo Fischer Scientific) as described above.

### 4.8. Chromatin Immunoprecipitation and RNA Immunoprecipitation

Chromatin immunoprecipitation (ChIP) and RNA immunoprecipitation (RIP) analyses were carried out on normoxic and hypoxic EA.hy926 cells (~1 × 10^7^ cells) for strong enhancer marks H3K27ac (Abcam, Cambridge, UK) and H3K9ac (Cell Signaling Technologies, Leiden, The Netherlands). Cross-linking, cell lysis and sonication were performed as described in the ChIRP protocol. After antibody incubation overnight, ChIP-grade Protein A/G magnetic beads (Thermo Fisher Scientific) were added for 2 h. Beads were washed with low-salt wash buffer (0.1% SDS, 1% Triton X-100, 2 mM EDTA, 20 mM Tris-HCl (pH 8.1), 150 mM NaCl), high-salt wash buffer (0.1% SDS, 1% Triton X-100, 2 mM EDTA, 20 mM Tris-HCl (pH 8.1), 500 mM NaCl), LiCl wash buffer (0.25 M LiCl, 1% NP40, 1% sodium deoxycholate, 1 mM EDTA, 10 mM Tris-HCl (pH 8.1)) and TE (Tris + EDTA) buffer (10 mM Tris-HCl, 1 mM EDTA (pH 8.0)). For ChIP, DNA was eluted with ChIP elution buffer (1% SDS, 0.1 M NaHCO3) at 65 °C for 10 min. Elute was treated with 0.5 M NaCl, 50 µg/mL RNase A (Thermo Fisher Scientific) and 100 µg/mL Proteinase K (NEB) at 65 °C overnight. DNA was purified with QIAquick PCR Purification Kit (Qiagen) and the relative enrichment of the epigenetic marks was determined at the *VEGF-AS1* and *VEGF-AS2* loci using qPCR (Supplementary Materials Table S2). For RIP, beads were heated at 95 °C for 10 min and RNA was extracted with TRIzol (Thermo Fischer Scientific) as described above.

## Supplementary Materials

The following are available online at http://www.mdpi.com/2311-553X/4/4/34/s1, Figure S1: *VEGF-AS1* and *VEGF-AS2* localize to the *VEGF-A* promoter in hypoxia; Figure S2: Repression of *VEGF-AS1* results in the downregulation of *VEGF-A* expression in PC-3 cells; Table S1: The sequences of *RP1-261G23.7* and *EST AV731492;* Table S2: Oligonucleotide and primer sequences.
