# Supplementary material for: Long Non-Coding RNA Modulation of VEGF-A during Hypoxia
Source: Noncoding RNA. 2018 Nov 20;4(4):34. doi: 10.3390/ncrna4040034 (PMC6315885; doi:10.3390/ncrna4040034)
Supplement: Supplementary file 1 [file ncrna-04-00034-s001.pdf]

Long non-coding RNA modulation of VEGF-A during hypoxia

Tiina Nieminen, Tristan A. Scott, Feng-Mao Lin, Zhen Chen, Seppo Yla-Herttuala and Kevin V. Morris

Table S1

Sequence of RP1-261G23.7 cloned into pcDNA3.1.

5'GTCGCTTTCGCTGCTCGCACGCCCGCGCGCTCTCTCTGACCCCGTCTCTCTCTTCCTCG
ACTTCTCTCTGGAGCTCTTGCTACCTCTTTCCTCTTTCTGCTGGTTTCCAAAATCCACAGTG
ATTTGGGGAAGTAGAGCAATCTCCCCAAGCCGTCGGCCCGATTCAAGTGGGGAATGGCAA
GCAAAAA3'

Sequence of EST AV731492 cloned into pcDNA3.1.

5'GAGAGAGGGCAGGGCAGAGTCACTGTCTGGGGAAGCTCACCTACCCACACCACCCCTA
GGCCCATCAGCCTCTGCCTCTCCTTCATGGGCCCCAGTCTAATCCCCTGACACTAAGCTGT
CCCTGATGAATTGCATCTCCCCAACACCCCCAGGCATGCTCACCTTCTGACACTCCAATCT
CTGCTATTCCTCCAAGTCTGACCCCAAAACATGCCCATTTCTGCCCCTGTTCACATCCTCCA
CCCCTACCCTGTCTTGGAGGGAGTCCTTCTGCTGGGAAGAAGGTATGGCATGTGGTGGCA
ACTAAGGTCCCAAAGCTGTATCTGCCCTTTTGACCTGGGCCTTGCCTGAGAGCTGAGCTAA
GCTCCAATTTGTGAATATTTGTAGGGACTACCAGGCACCAGGCTCCATGCTGATTTTATAG
GAGGTACAGGGACATCTAGGTAGGGCAGTGGAAAGTACAGGGACTTTGGATTCAAATGGA
CCCAAGTCACATTCCTGGCTCTGCCACTCACTGTGTGGCCTTAGGTTATTCAACCTCTCTG
AGCCACAGCTCCCCTCATCAAAAATAGGAGTAAAAATCCCTGCCTTAGAGAGTTGCTGCAC
ACAGACAAATGTGCACACAGCTGCCCCAGAAAGCAAGATGAGAAAGAGGCCCTGGGGGA
GGCACAAGTGTTGTG3'

Table S2

Oligonucleotide and primer sequences

Name	Sequence (5'-3')	Function
sgRNA2.1 (+)	GATCCGAGGAGGGTAACTCTCCCAAG	Targets VEGF-AS2 putative promoter
sgRNA2.1 (-)	AAACCTTGGGAGAGTTACCCTCCTCG	Targets VEGF-AS2 putative promoter
sgRNA2.2 (+)	GATCCGGATCTGGAGGTTTGACTTG	Targets VEGF-AS2 putative promoter

sgRNA2.2 (-)	AAACCAAGTCAAACCTCCAGATCCG	Targets VEGF-AS2 putative promoter
sgRNA2.3 (+)	GATCCGCAATTCCCTGACCCTGACTG	Targets VEGF-AS2 putative promoter
sgRNA2.3 (-)	AAACCAGTCAGGGTCAGGGAATTGCG	Targets VEGF-AS2 putative promoter
sgRNA control (+)	GATCCGTCCCCAGTGCACACAGACCT	Targets Alpha-1 antitrypsin (no PAM sequence in the target site)
sgRNA control (-)	AAACAGGTCTGTGTGCACTGGGGACG	Targets Alpha-1 antitrypsin (no PAM sequence in the target site)
VEGF-AS1 PTO	A*G*A*G*G*A*A*A*G*A*G*G*T*A*G*C*A *A*G*A*G*C*T*C	Phosphorothioate oligonucleotide for VEGF-AS1 knockdown
VEGF-AS2 PTO	T*C*T*T*G*C*T*T*T*C*T*G*G*G*G*C*A* G*C*T*G*T*G*T	Phosphorothioate oligonucleotide for VEGF-AS2 knockdown
VEGF-S2 PTO	G*G*A*G*G*C*A*C*A*A*G*T*G*T*T*G*T* G*A*A*G*G*T*A	Phosphorothioate oligonucleotide for VEGF-S2 knockdown
Control PTO	A*C*T*G*A*C*C*T*T*T*G*G*A*T*G*G*T* G*C*T*A*C*A*A	Phosphorothioate oligonucleotide for miRN367 knockdown
AS1 Biotin 1	GTGCGAGCAGCGAAAGCGAC	3'Biotin oligonucleotides for IP of VEGF-AS1
AS1 Biotin 2	AGCAGAAAGAGGAAAGAGGT	3'Biotin oligonucleotides for IP of VEGF-AS1
AS1 Biotin 3	TTGCCATTCCCCACTTGAAT	3'Biotin oligonucleotides for IP of VEGF-AS2
AS2 Biotin 1	TAGTGTCAGGGGATTAGACT	3'Biotin oligonucleotides for IP of VEGF-AS2
AS2 Biotin 2	ATACAGCTTTGGGACCTTAG	3'Biotin oligonucleotides for IP of VEGF-AS2
AS2 Biotin 3	GTACCTCCTATAAAATCAGC	3'Biotin oligonucleotides for IP of VEGF-AS2
AS2 Biotin 4	CTTTCTCATCTTGCTTTCTG	3'Biotin oligonucleotides for IP of VEGF-AS2
VEGF-AS1 RT	CTTGCCATTCCCCACTTG	Gene-specific RT
VEGF-AS1 F	TCTCTGACCCCGTCTCTCTC	VEGF-AS1 expression
VEGF-AS1 R	CTTGCCATTCCCCACTTG	VEGF-AS1 expression
AS1 Target F	ACTTCCCCAAATCACTGTGG	VEGF-AS1 localization
AS1 Target R	GTCACTCACTTTGCCCCTGT	VEGF-AS1 localization

Set1a_F	TTTTGCTTGCCATTCCCCAC	Primer walking
Set1b_F	GGCTTGGGGAGATTGCTCTA	Primer walking
Set 1c_F	ACTTCCCCAAATCACTGTGG	Primer walking
Set1abc_R	GTCACTCACTTTGCCCCTGT	Primer walking
VEGF-AS2 RT	CCAGGGCCTCTTTCTCATCT	Gene-specific RT
VEGF-AS2 F	TGTGGTGGCAACTAAGGTCC	VEGF-AS2 expression
VEGF-AS2 R	CAGGAATGTGACTTGGGTCCA	VEGF-AS2 expression
AS2 Target F	TGTGACTTGGGTCCATTTGA	VEGF-AS2 localization
AS2 Target R	GGTCCCAAAGCTGTATCTGC	VEGF-AS2 localization
Set2a_F	CCTTCCAGATGCCCATTCTA	Primer walking
Set2a_R	ACAGCTCCCCTCATCAAAAA	Primer walking
Set2b_F	TGTGACTTGGGTCCATTTGA	Primer walking
Set2b_R	GGTCCCAAAGCTGTATCTGC	Primer walking
Set2c_F	GCATGTTTTGGGGTCAGACT	Primer walking
Set2c_R	AGCTCACCTACCCACACCAC	Primer walking
VEGF-S2 RT	TAGCCCCATGTGGATCTGGA	Gene-specific RT
VEGF-S2 F	CCTTCCAGATGCCCATTCTA	VEGF-S2 expression
VEGF-S2 R	ACAGCTCCCCTCATCAAAAA	VEGF-S2 expression
Spliced VEGF-A_ F	CCCACTGAGGAGTCCAACAT	VEGF-A expression(Exon 3/6)
Spliced VEGF-A_R	TTTCTTGCGCTTTCGTTTTT	VEGF-A expression(Exon 3/6)
Unspliced VEGF-A_F	AGGGAAAGGGGCAAAAACGA	VEGF-A unspliced (Exon6/intron)
Unspliced VEGF-A_R	GAGGATGAGAGCCAGGGAAG	VEGF-A unspliced (Exon6/intron)
B2M_F	TAGAGGTGGGGAGCAGAGAA	Endogenous control expression
B2M_R	TCCCCCAAATTCTAAGCAGA	Endogenous control expression
Off-target F	GGGGCTTTCAAGGTAACTCC	Off-target site for localization

Off-target R	AGGGCCCCATAGAGAAGAGA	Off-target site for localization
NEAT1_F	AGCTGGAAGTCTTAGAAAAGCCT	NEAT1 expression
NEAT1_R	ACAGATGTGTTTCTAAGGCACG	NEAT1 expression
5C2	GATGCCCATTCTAGCCAGTC	sgRNA for VEGF-AS2 KO cell line
3C2	AGGAGGGTAACTCTCCCAAG	sgRNA for VEGF-AS2 KO cell line
VEGF-AS1 PTO a	A*G*A*G*G*A*A*A*G*A*G*G*T*A*G*C*A *A*G*A*G*C*T*C	Phosphorothioate oligonucleotide for VEGF-AS1 knockdown in PC-3
VEGF-AS1 PTO b	A*T*T*T*T*G*G*A*A*A*C*C*A*G*C*A*G* A*A*A*G*A*G*G	Phosphorothioate oligonucleotide for VEGF-AS1 knockdown in PC-3
GAPDH Biotin 1	ATGGTACATGACAAGGTGCG	3'Biotin oligonucleotides for IP of GAPDH (Control)
GAPDH Biotin 2	ATACCAAAGTTGTCATGGATGA	3'Biotin oligonucleotides for IP of GAPDH (Control)

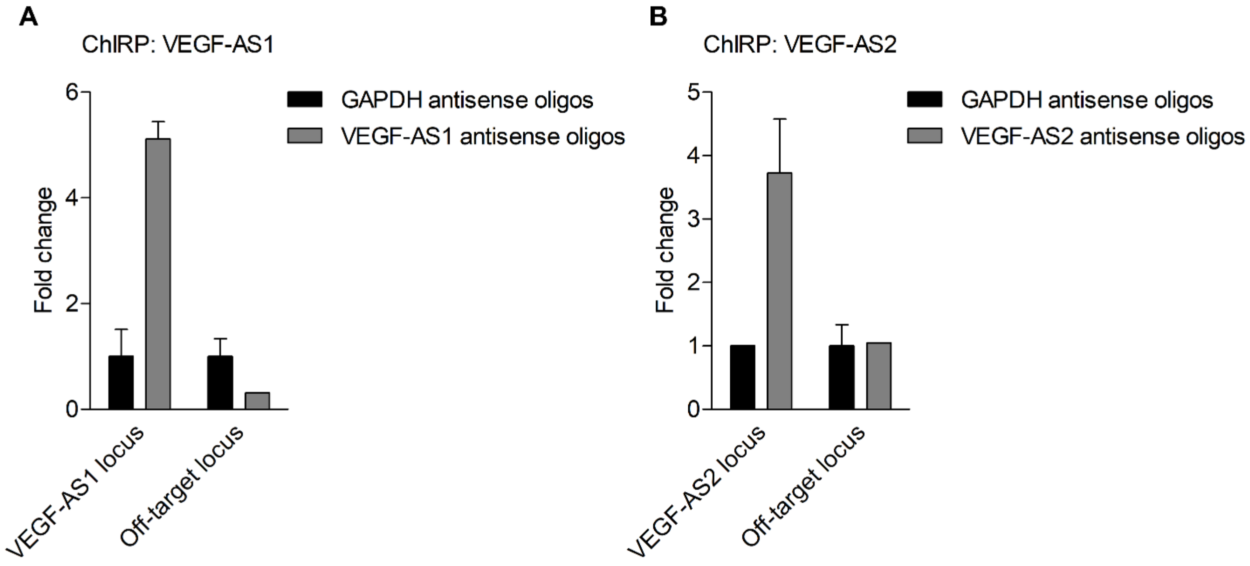

Figure S1. VEGF-AS1 and VEGF-AS2 localize to the VEGF-A promoter in hypoxia. (**a**) Fold change in VEGF-AS1 target locus enrichment at the VEGF-A promoter in hypoxic EA.hy926 cells as determined by qPCR after pulldown with antisense oligonucleotides with 3'-Biotin modifications followed by RNase A and RNase H treatments. The data are presented as mean ± SD and standardized to inputs; (**b**) Fold change in VEGF-AS2 target locus enrichment at the VEGF-A promoter in hypoxic EA.hy926 cells as determined by qPCR after pulldown with antisense oligonucleotides with 3'-Biotin modifications followed by RNase A and RNase H treatments. The data are presented as mean ± SD and standardized to inputs.

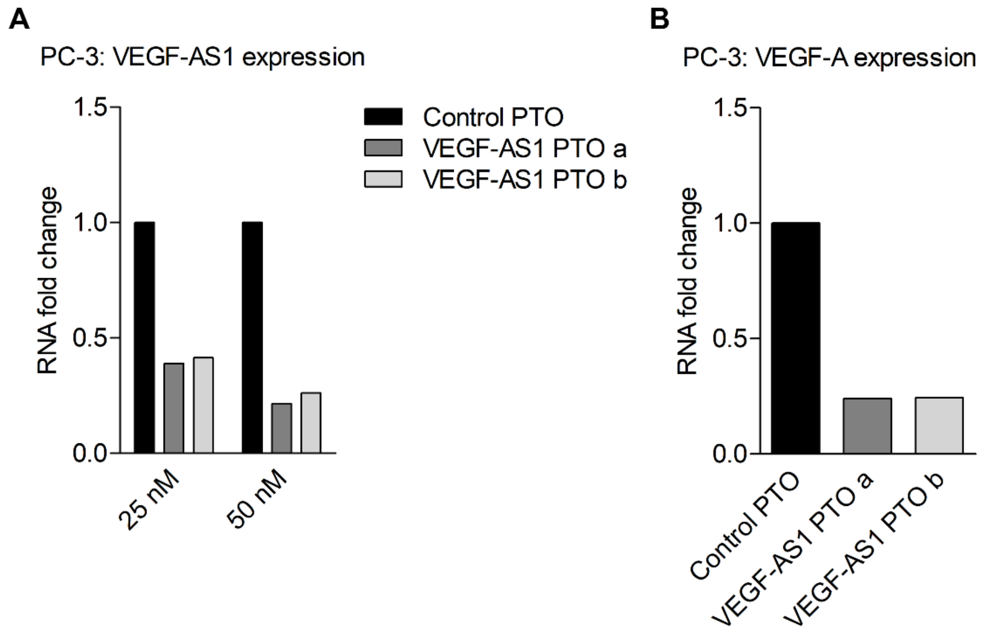

Figure S2. Repression of VEGF-AS1 results in the downregulation of VEGF-A expression in PC-3 cells. (**a**) Fold change in VEGF-AS1 expression levels in normoxic PC-3 cells 48h after antisense PTO transfections as determined by qRT-PCR and standardized to B2M; (**b**) Fold change in spliced VEGF-A expression levels in normoxic PC-3 cells 48h after antisense PTO transfections (50 nM) as determined by qRT-PCR and standardized to B2M.
